# Weight Velocity in Addition to Latest Weight Does Not Improve the Identification of Wasting or the Prediction of Stunting and Mortality: A Longitudinal Analysis Using Data from Malawi, South Africa, and Pakistan

**DOI:** 10.1016/j.tjnut.2024.06.011

**Published:** 2024-06-25

**Authors:** Charlotte M Wright, Fanny Petermann-Rocha, Ruth Bland, Per Ashorn, Shakila Zaman, Frederick K Ho

**Affiliations:** 1 School of Medicine, Dentistry & Nursing, University of Glasgow, Glasgow, United Kingdom; 2 Centro de Investigación Biomédica, Facultad de Medicina, Universidad Diego Portales, Santiago, Chile; 3 School of Medicine, University of Glasgow, Glasgow, United Kingdom; 4 Center for Child Health Research, Tampere University and Tampere University Hospital, Tampere, Finland; 5 Department of Public Health, University of Health Sciences, Lahore, Pakistan; 6 School of Health and Wellbeing, University of Glasgow, Glasgow, United Kingdom

**Keywords:** children, mortality, growth disorders, stunting, weight

## Abstract

**Background:**

In low/middle-income countries, most nutritional assessments use the latest weights, without reference to growth trajectory.

**Objectives:**

This study explores whether velocity, in addition to the latest weight, improves the prediction of wasting, stunting, or mortality in the first 2 years of life.

**Methods:**

We analyzed a combined data set with weight and height data collected monthly in the first year of 3447 children from Pakistan, Malawi, and South Africa, with height and survival recorded till 24 m. The main exposures were weight-for-age z-score (WAZ) at the end of each 2-m period and weight velocity-for-age z-score (WVZ_2_) across that period. The outcomes were wasting, stunting, or all-cause mortality in the next 1–2 mo. As a sensitivity analysis, we also used WVZ over 6 mo (WVZ_6_), with matching WAZ. Cox proportional hazard models with repeated growth measures were used to study the association between exposures and mortality. Mixed Poisson models were used for stunting and wasting.

**Results:**

Children who were already stunted or wasted were most likely to remain so. Higher WVZ_2_ was associated with a lower risk of subsequent stunting (risk ratio [RR]: 0.95; 95% confidence interval [CI]: 0.93, 0.96), but added minimal prediction (difference in AUC = 0.004) compared with a model including only WAZ. Similarly, lower WVZ_2_ was associated with wasting (RR: 0.74; 95% CI 0.72, 0.76) but the prediction was only marginally greater than for WAZ (difference in AUC = 0.015). Compared with WAZ, WVZ_6_ was less predictive for both wasting and stunting. Low WVZ_6_ (but not WVZ_2_) was associated with increased mortality (hazard ratios: 0.75, 95% CI: 0.67, 0.85), but added only marginal prediction to a model including WAZ alone (difference in C = 0.015).

**Conclusions:**

The key anthropometric determinant of impending wasting, stunting, and mortality appears to be how far below the normal range the child’s weight is, rather than how they reached that position.

## Introduction

Child undernutrition remains a major global health concern [1]. WHO definitions of malnutrition rely on single measures of weight and length/height and, more recently, mid-upper arm circumference, as well as the combination of weight and length/height to give weight-for-height. The most common measure is that of weight, usually adjusted for age via a lookup table, plotting on a chart, or converting to a standard deviation (z) score. A weight-for-age z-score (WAZ) <–2 is defined as moderate malnutrition and <–3 as severe, whereas a length-for-age z-score (LAZ) <–2 is defined as stunting [2]. Regularly weighing aims to identify children who have lost or gained weight more slowly than their peers and are malnourished, so a measure that determines children with low weight velocity (WV) might be expected to be more discriminating than attained weight (AW) alone. In high-income countries, this has led to the concept of failure to thrive or weight faltering, where the diagnosis depends on low WV based on 2 or more weights, at least while a child is within the normal range [3].

In contrast, in low- and middle-income countries, decisions tend to be based only on the current weight, as interpretation of serial measurements require more detailed record-keeping and WHO definitions of malnutrition include no velocity elements [4]. However, the WHO growth chart project has published age and sex-standardized norms for weight velocity over 4- and 8-wk intervals [5], and they have argued that velocity measures are helpful in predicting later stunting [6]. As severe malnutrition (SAM) becomes less common [7], the accurate diagnosis of moderate malnutrition (MAM) becomes more important, to target treatment to children who are most likely to benefit. Using only a low AW will also identify children who were born small but have grown steadily since birth and supplementary feeding could even harm these children [8]. Thus, a secondary screening measure of velocity could potentially be used to improve the specificity of diagnoses.

Few studies have examined whether velocity improves the prediction of adverse growth outcomes, compared with using a single weight [7,8]. Only one has considered outcomes beyond the age of 6 mo or used increments >1 mo [9]. Thus, it is unclear whether velocity adds value in addition to the latest weight alone, and if so, when, and over what time interval weight velocity might usefully be measured.

We thus aimed to use a large combined historical data set of longitudinal growth data, with a high prevalence of malnutrition, to examine the value of WV, in addition to AW, in identifying the onset of wasting and predicting stunting or mortality at different ages, and over differing time intervals.

## Methods

This analysis used data from 3 prospective cohort studies conducted in low or middle-income countries, all with monthly measurements collected for at least the first 12 mo and outcome of interest ≤24 mo. These were the Lungwena Child Survival Study (Malawi), the Africa Center Vertical Transmission Study (South Africa), and the Lahore longitudinal study (Pakistan). Each study is briefly described below.

### Included cohort studies

The Lungwena Child Survival Study was a cohort study of 795 women recruited between 1995 and 1996 and their newborn children prospectively studied [10,11]. Children were measured monthly to 18 mo, then every 3 mo ≤36 mo. Children were measured at home by a research assistant using portable spring scales and length boards [12].

The Africa Center Vertical Transmission Study registered 2938 children (half HIV-positive) from 7 rural, 1 semi-urban, and 1 urban primary health care clinic in KwaZulu-Natal, in a nonrandomized intervention cohort study between 2001 and 2005 [13,14]. Weight and lengths were collected by research staff, using the WHO-recommended protocol, monthly until 12 mo, then every 3 months–24 mo [12].

The Lahore longitudinal study enrolled infants born between 1984 and 1994, with 1314 from a village area, 572 from a periurban slum, 921 from an urban slum, and 339 from a middle-class neighborhood, and followed them monthly from birth ≤36 mo [15,16]. All infants were weighed and measured at home by specially trained research assistants, with the measuring technique checked monthly and instruments checked weekly [12,17].

In the 3 studies, all deaths were recorded, but their causes were not recorded consistently between datasets. Sociodemographic information (age, sex, and country of origin) was self-reported. HIV-positive mothers and subsequent HIV-positive children were excluded from the analyses.

### Statistical analyses

The 3 datasets were combined into 1 database (excluding all HIV-positive mothers) and measurements were expressed as z-scores compared with the WHO growth standard [18]. Stunting was defined as LAZ <-2 SD and wasting as weight-for-length (WLZ) <–2. The analysis was then conducted per measurement rather than per child. For each AW, the exposures of interest were the WAZ and the weight velocity z-score (WVZ) across the period up to that AW, calculated using the WHO velocity standards [5] for both 2- and 6-mo intervals. The outcome was whether or not the child was stunted or wasted at the next monthly measurement, or whether they had died. Attained WLZ and LAZ were treated as secondary or additional exposures.

Prediction models were constructed using weight velocity and the attained growth parameters at the end of the weight velocity period, to predict the next observation of wasting, stunting, or mortality. Because each growth measurement was used to predict the subsequent period’s wasting and stunting risk, mixed Poisson models with repeated growth measures were used to study the associations. All observations were included in the model to maximize power while intra-personal correlations were captured as a random intercept. Results are reported as risk ratios (RRs) with their respective 95% CI. Model predictive performance was assessed with the area under the receiver operating characteristic curve. The main analyses were adjusted for age at assessment, sex, and country of origin. In addition, analyses were stratified for incident and recurrent wasting/wasting for the predictors that had the highest AUC.

Cox proportional hazard models with repeated growth measures were used to study the association between exposures and mortality rate. The outcome variable was time to event (either death or censoring). The basic analyses were adjusted for age at assessment, sex, and country of origin (model 1). Additional models included the mutual adjustment among the growth parameters. Analyses are reported as hazard ratios (HRs) with 95% confidence intervals (Cis). In the mortality analysis, the predictive ability was quantified using Harrell’s C-index—which estimates the probability of concordance between observed and predicted responses [19].

Finally, sensitivity analyses were used to investigate whether the associations observed differed by age (≤ and >6 mo). The interaction between the binary age group (≤ compared with >6 mo) and weight variables were included in the corresponding regression models and can be interpreted as the ratio of RR (for wasting and stunting) and the ratio of HR (for mortality). A ratio >1 indicates that the association of the weight variable with the outcome is stronger in the older (>6 mo) group.

R 4.0.5 software was used to perform all analyses. A *P* value of ≤0.05 was considered statistically significant.

## Results

**FIGURE 1:**
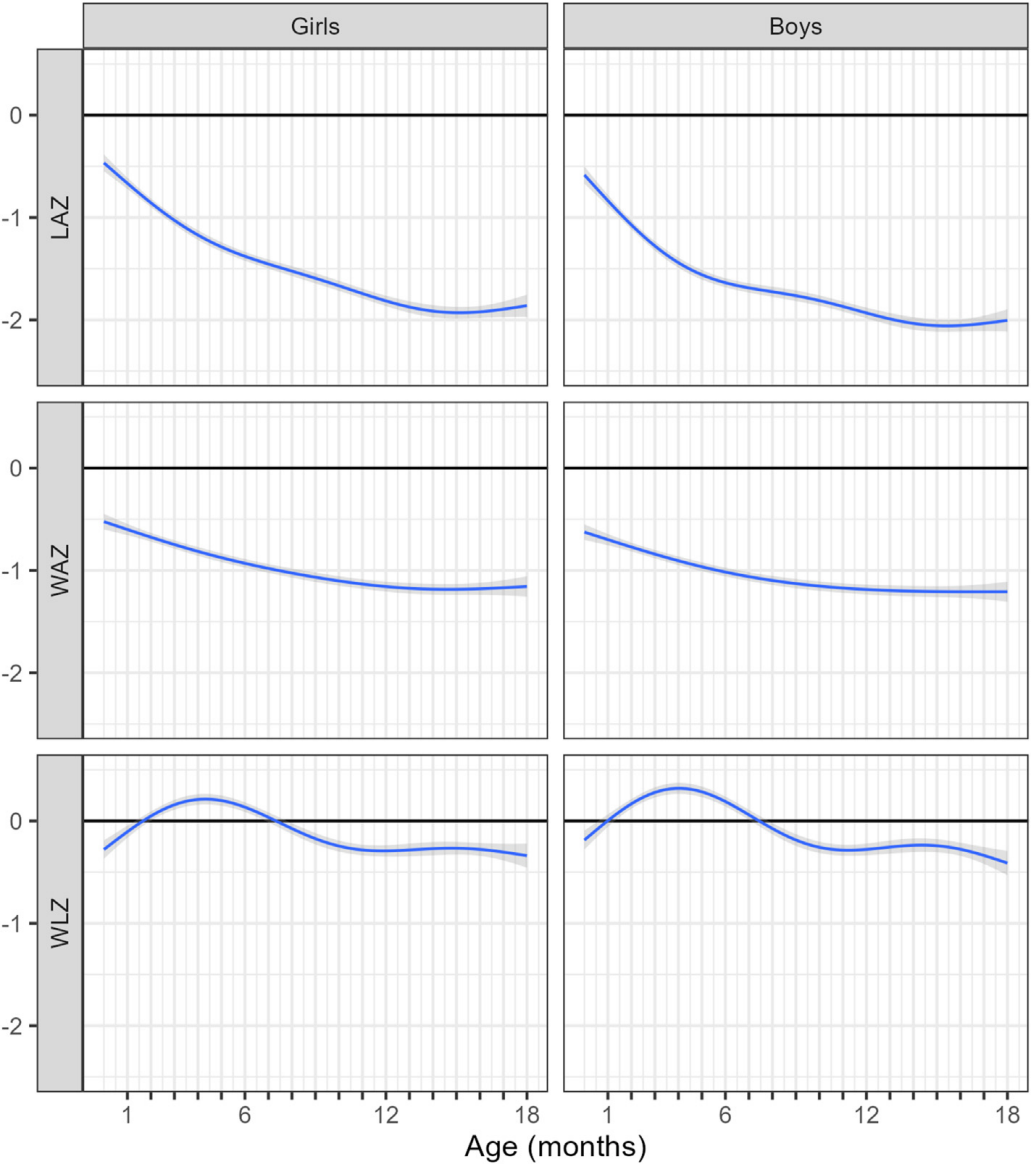
Age trend of growth indicators by sex. LAZ, length-for-age z-score; WAZ, weight-for-age z-score; WLZ, weight-for-length z-score.

The baseline characteristics of the included data are shown in Table 1. Of the 3447 children with any pairs of growth data, 98 children died, and in a third of these, this followed a weight <–2 SD. Of the included weights, the majority (78%) were within the normal range, but 20% were <–2 (underweight) and only 1% were >2. Using the first measurements available near birth, 11.8% of children were <–2 WAZ and 14.4% <–2 LAZ. The majority of underweight measures came from the Pakistan periurban and village cohorts. On average children were already below expected length by age 1 mo and dropped to an average 2 SD below the mean by the second year of life, 14% were already <–2 LAZ (Figure 1). Their weight showed a similar but less extreme pattern. Weight-for-length rose in the first few weeks and remained close to expected levels for the first 6 mo, then dropped slightly below thereafter.

**TABLE 1:** Baseline characteristics per attained weight WAZ categories

	Baseline WAZ categories		
	<–2	–2 to 2	>2
Total number of measurements, *n* (%)	4167 (20.4)	15,953 (78.3)	265 (1.3)
No. (%) in each WAZ category by cohort:			
Malawi	842 (20.2)	2734 (17.1)	4 (1.5)
South African and Pakistan middle class	154 (3.7)	3968 (24.9)	201 (75.9)
Pakistan periurban and village	2303 (55.3)	4595 (28.8)	0
Pakistan urban slum	868 (20.8)	4656 (29.2)	60 (22.6)

Abbreviations: *n*: number; WAZ: weight-for-age z-score.

The associations of WAZ and WVZ with subsequent wasting and stunting are shown in Table 2. For both wasting and stunting, after adjustment for age, sex, country of origin, and already being wasted or stunted, respectively, weight velocity z-score over 2 mo (WVZ_2_) was a stronger predictor than weight velocity z-score over 6 mo (WVZ_6_), but both were lower than for WAZ alone. The model including WVZ_2_ as well as WAZ had marginally higher prediction performance for subsequent wasting (AUC gain 1.5%) than the model with only WAZ, but for predicting stunting: the model including WAZ and WVZ_2_ the AUC was almost unchanged (AUC gain 0.4%).

**TABLE 2:** Associations of attained weight and weight velocity in 1 period, with wasting and stunting in the next period

Predicting wasting in the next period	RR (95% CI)	AUC	% change in AUC vs REF	
Model 1		0.872 (0.861–0.884)	REF	
Wasting in this period	9.91 (8.3, 12.8)			
Model 2		0.916 (0.908–0.924)	4.5	REF
Wasting in this period	3.84 (3.1, 4.7)			
WAZ	0.66 (0.6, 0.7)			
Model 3		0.888 (0.878–0.899)	1.7	
Wasting in this period	11.8 (10.5, 13.2)			
WVZ_2_	0.76 (0.74, 0.79)			
Model 4		0.869 (0.858–0.880)	–0.3	
Wasting in this period	11.8 (10.5, 13.2)			
WVZ_6_	0.83 (0.81, 0.86)			
Model 5		0.931 (0.927–0.941)	6.0	1.5
Wasting in this period	4.06 (3.4, 4.9)			
WAZ	0.62 (0.58, 0.66)			
WVZ_2_	0.74 (0.72, 0.76)			
Predicting stunting in the next period				
Model 1		0.910 (0.905–0.915)	REF	
Stunting in this period	6.61 (6.2, 7.0)			
Model 2		0.929 (0.925–0.934)	2.0	REF
Stunting in this period	5.30 (4.9, 5.7)			
WAZ	0.86 (0.85, 0.88)			
Model 3		0.913 (0.909–0.918)	0.3	
Stunting in this period	6.62 (6.2, 7.1)			
WVZ_2_	0.96 (0.95, 0.97)			
Model 4		0.912 (0.907–0.917)	0.2	
Stunting in this period	6.63 (6.2, 7.1)			
WVZ_6_	0.96 (0.95, 0.98)			
Model 5		0.933 (0.929–0.937)	2.3	0.4
Stunting in this period	5.22 (4.99, 5.6)			
WAZ	0.85 (0.84, 0.87)			
WVZ_2_	0.95 (0.93, 0.96)			

Abbreviations: AUC, area under the curve; CI, confidence interval; RR, risk ratio; WAZ: weight-for-age z-score; WVZ_2_: Weight velocity z-score 2-mo interval. WVZ_6_: Weight velocity z-score 6-mo interval.

Data presented as risk ratios (RRs) and their 95% CI using linear mixed-effect models. All models were adjusted for age, sex, and country of origin and then mutually adjusted for all variables listed. All relative risks were *P* < 0.001.

The associations for those who were not wasted or stunted at the time of the attained WAZ (incident cases) and for children who were already stunted or wasted are shown in Table 3. This revealed that, although both WAZ and WVZ_2_ were more predictive of incident cases than persistence in prevalent cases, WVZ essentially added no extra prediction of incident cases. It did, however, increase the prediction of recovery from wasting (change in AUC 9.8%) but only marginally from stunting (change in AUC 1.4%).

**TABLE 3:** Associations of attained weight and weight velocity with subsequent wasting and stunting in incident and prevalent existing cases

	Risk of incident wasting in nonwasted children			Risk of continued wasting in already wasted children		
	RR (95% CI)	*P* value	AUC	RR (95% CI)	*P* value	AUC
Predicting wasting						
Model with WAZ only	0.49 (0.45, 0.53)	<0.001	0.912 (0.902–0.922)	0.84 (0.80, 0.88)	<0.001	0.707 (0.683–0.733)
Model WVZ_2_ only	0.70 (0.70, 0.70)	<0.001	0.916 (0.604–0.928)	0.83 (0.79, 0.86)	<0.001	0.743 (0.718–0.769)
Model with both			0.914 (0.902–0.925)			0.805 (0.783–0.828)
WAZ	0.46 (0.42, 0.50)	<0.001		0.83 (0.79, 0.87)	<0.001	
WVZ_2_	0.67 (0.64, 0.70)	<0.001		0.82 (0.79, 0.86)	<0.001	
Gain in AUC by adding WVZ to the model			0.2%			9.8%
	Risk of incident stunting in nonstunted children			Risk of continued stunting in already stunted children		
Model with WAZ only	0.52 (0.49, 0.55)	<0.001	0.837 (0.826–0.848)	0.94 (0.92, 0.96)	<0.001	0.761 (0.742–0.780)
Model WVZ_2_ only	0.89 (0.86, 0.91)	<0.001	0.832 (0.820–0.844)	0.99 (0.97, 1.00)	<0.001	0.678 (0.656–0.701)
Model with both			0.831 (0.825–0.847)			0.775 (0.757–0.793)
WAZ	0.49 (0.46, 0.52)	<0.001		0.93 (0.91, 0.96)	<0.001	
WVZ_2_	0.84 (0.81, 0.52)	<0.001		0.98 (0.97, 1.00)	0.02	
Gain in AUC by adding WVZ to model (%)			–0.1%			1.4%

AbbreviationsAUC, area under the curve; CI, confidence interval; RR, risk ratio; WAZ, weight-for-age z-score; WVZ, weight velocity z-score.

Data presented as risk ratios (RRs) and their 95% CI using linear mixed-effect models. The created groups were: people who had wasting/stunting at baseline (yes/no) vs. those who developed later (yes/no). The main analyses were adjusted for age, sex, country of origin, and wasting/stunting status in the last period.

Associations of growth parameters with mortality are shown in Table 4. Adjusted just for age, sex, and country of origin (model 1), WAZ, WLZ, and WVZ (6-mo interval) were separately associated with lower mortality risk, whereas WVZ (2-mo interval) and LAZ were not significantly associated with mortality. Among combinations of the 3 significant predictors, the combination of WAZ and WVZ (6-mo interval) yielded the strongest prediction (C-index 0.8842), slightly greater than using WAZ (C-index 0.8647) or WVZ-6 alone (0.8718) but the overall gain was still only 2%.

**TABLE 4:** Associations of attained height, weight, and weight velocity with mortality

	HR (95% CI)	*P* value	C-index (95% CI)	Change in C-index (%)
Each variable is added singly				
WAZ = model 1	0.84 (0.73, 0.98)	0.023	0.8647 (0.8263, 0.9032)	REF
WLZ	0.83 (0.72, 0.97)	0.02	0.8626 (0.8234, 0.9018)	–0.2
LAZ	0.91 (0.77, 1.08)	0.30	0.8592 (0.8203, 0.8980)	–0.6
WVZ (2-mo interval)	0.93 (0.80, 1.06)	0.30	0.8606 (0.8226, 0.8985)	–0.4
WVZ (6-mo interval)	0.78 (0.69, 0.88)	<0.001	0.8718 (0.8325, 0.9110)	0.7
Model 2 (mutually adjusted)			0.8842 (0.8479, 0.9205)	2.0
WAZ	0.83 (0.72, 0.96)	<0.001		
WVZ (6-mo interval)	0.78 (0.69, 0.88)	<0.001		
Model 3 (mutually adjusted)			0.8794 (0.8426, 0.9162)	1.5
WLZ	0.77 (0.66, 0.89)	<0.001		
WVZ (6-mo interval)	0.75 (0.67, 0.85)	<0.001		
Model 4 (mutually adjusted)			0.8655 (0.8270, 0.9041)	0.1
WAZ	0.92 (0.74, 1.13)	0.40		
WLZ	0.89 (0.72, 1.10)	<0.001		
Model 5 (mutually adjusted)			0.8801 (0.8435, 0.9167)	1.5
WAZ	0.98 (0.80, 1.21)	0.90		
WLZ	0.78 (0.63, 0.96)	0.02		
WVZ (6-mo interval)	0.75 (0.67, 0.85)	<0.001		

Abbreviations: CI, confidence interval; HR, hazard ratio; LAZ, length-for-age z-score; WAZ, weight-for-age z-score; WLZ, weight-for-length z-score; WVZ, weight velocity z-score.

Data presented as hazard ratios (HRs) with their respective 95% CI using Cox proportional models. All analyses were adjusted for age, sex, and country of origin. Total measurements: 20,385 and deaths: 98. The C-index is an indicator of risk prediction which estimates the probability of concordance between observed and predicted responses. Values close to 0.5 are equivalent to a random guess, whereas 1.0 is equivalent to perfect prediction.

Supplemental Tables 1–3 show the analysis of potential interaction with age group. On predicting wasting, in the combined model, WAZ showed a 12% increased association (ratio of RR= 0.88; 95% CI: 0.81, 0.95) in older children, whereas WVZ-2 was 6% greater (ratio of RR: 1.06; 95% CI: 1.01, 1.12). There was no evidence for age interactions for predicting stunting and mortality.

## Discussion

This study used data from 3 cohort studies to describe the extent to which recent changes in weight (velocity) added to the accuracy of prediction of later wasting, stunting, and mortality, compared with a single weight alone. Surprisingly, combining weight velocity with AW added little or no additional predictive value, except in predicting whether already wasted children would recover.

This finding is in keeping with the limited number of other papers that have directly compared velocity with single measures, mostly in relation to mortality. Two early studies found that AW and height for age performed better than weight or height velocity as discriminators of mortality [20,21], while a more recent article found little difference between various single measures and velocity [22]. One group has found velocity to be a stronger predictor of mortality in 1 data set [23], but in a second set, they found no increased prediction when adding velocity [24]. In another study, the same group also considered predicting wasting and stunting and found that velocity increased prediction up to age 2 mo [24] but not at later ages [9].

There are many possible reasons why velocity may not be a useful discriminator. Velocity is vulnerable to error as it requires 2 measurements, both with a degree of measurement error (for example, instrument imprecision and differences in procedures) plus the variability of weight over short intervals due to ingestion and elimination [25]. Although these data were collected as part of research studies, they were largely collected in real-life situations where one could speculate that at 1 visit a young child might have just eaten and drunk largely, whereas at the next they might have fasted or just emptied their bowels or bladder. These combined are thus inevitably less accurate than a single weight measurement. Thus, a child could show a large meaningless variation (“noise”), from 1 time to the next that has no long-term significance, but has the potential to drown out true meaningful variation—the “signal” [25].

It must also be born in mind that weight velocity is calculated between 2 measurements, and in utero growth could not be measured. If the decline in growth occurred in utero, the size at birth may be low, but there will be no observable decline in velocity. As a group, these children had a high prevalence of stunting, illustrated by the fact that the mean LAZ in the second year was close to –2 (that is, 2 SD below the mean). It has been well shown that a high proportion of stunting has already occurred very early in the first year [26]. Other studies have shown that in uncompromized environments most children with slow intrauterine growth will show rapid catchup in the first 4 mo [27], but in suboptimal circumstances, recovery from stunting is rare [26]. Thus most of these children remain at a similar centile, rather than showing a detectable decline. This may also apply to the onset of wasting; a previous study in young infants that compared AW with velocity and skinfolds as a measure of low-fat stores found that low weight was highly specific for both low velocity and low fat, but that only 40% of those with low fat had shown slow weight gain [28], suggesting that they had started life with low-fat stores and failed to acquire fat, rather than faltering.

**FIGURE 2:**
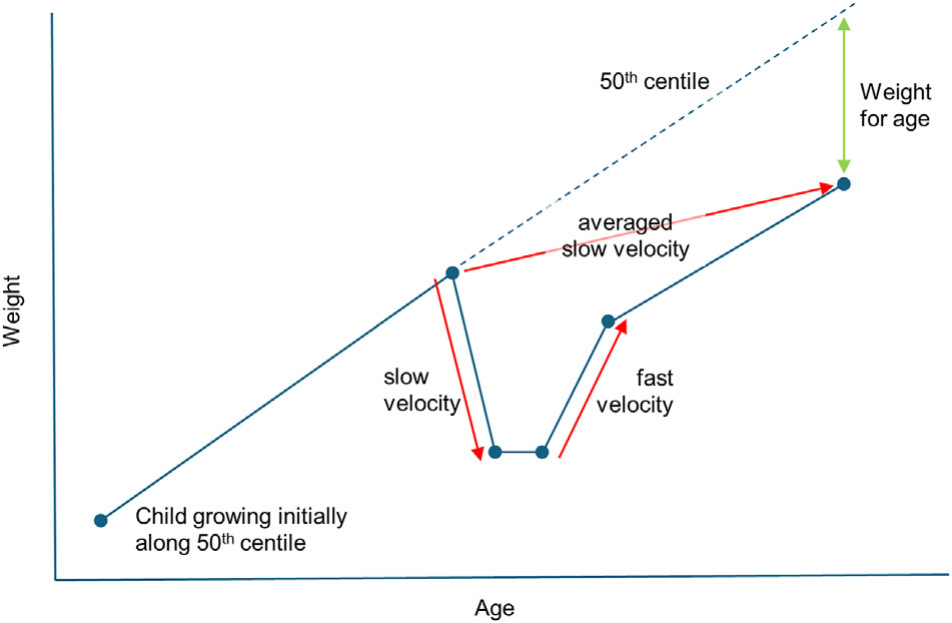
The challenge of assessing weight gain across an episode of weight faltering. A child showing slow, then fast velocity. This averages to a relatively slow velocity over a longer time, but with 2 sources of error. The last weight-for-age z-score describes how much the weight deviates from the average for that age, with only 1 source of error.

Finally, it should be recognized that velocity may vary rapidly over time, resulting in varying measurements depending on when the measurement was taken and the duration where velocity is calculated. A recent study collected daily weights in a group of Gambian infants used a spline curve to “smooth out minor day-to-day fluctuations” and found that short episodes of weight loss were common (mean 18 d), but these were followed by a similar period (mean 17 d) of catchup [29]. Depending on the times when weights were collected, the same child might thus be labeled as having very low or very high velocity (Figure 2). Over a longer period, the net effect may have been slower than expected weight gain, but it had 2 sources of errors. This deviation can equally be detected in most children simply using weight-for-age, as this describes how much the weight deviates from the average for that age, with only 1 error. So weight-for-age will always tend to be more accurate than the net velocity in initially average children, who will always be in the majority.

### Strengths and limitations

This study was able to combine data from 3 cohorts with comparable measurements, which enabled us to answer the research question with more events than analyzing each cohort separately. Nonetheless, this study is not without limitations. First, as a group, these children had very high rates of stunting, but our earlier description of this cohort showed that although incident wasting was higher than expected, it was much less common. [12]. So in this setting, variations in WAZ may be more likely to reflect slowing of growth rather than short-term weight loss. Data collection was carried out >20 y ago when mortality and SAM were generally more common in the area. Thus, these findings may not be representative of modern populations. We have not considered the potentially modifying role of diet, in particular duration of breastfeeding and age of first solids, environmental conditions, or socioeconomic conditions, as these were not consistently collected between data sets. Similarly, we have not considered the role of birth length and weight in the analysis, as this study focuses on the prediction value of weight velocity in practice, where birth length and weight are often not available.

### Implications

It turns out that the usual practice of concentrating on the current weight, is better than the proposed alternatives, as well as the easiest means of detecting wasting or predicting future adverse events. It is reassuring that this analysis found no justification for incorporating weight velocity measures in routine screening programs. These already struggle to find time to process even single weights and heights and incorporating the change from an earlier weight adds considerable complexity: requiring the earlier weight to be accessed and the change interpreted, although recent developments in digital support for growth monitoring have the potential to make the interpretation easier [30].

The 1 possible exception for this is in the assessment of children with moderate or severe acute malnutrition (as indicated by wasting in this study), where the recent growth pattern substantially improved the prediction of persistence or recovery. This may lend support to the practice in many nutrition clinics of monitoring weight gain over time, not just AW, although this is not recommended by WHO [4].

In conclusion, although hypothetically useful in detecting and predicting low growth and mortality, velocity measures add little or no predictive power, probably due to their increased imprecision compared with a single recent weight and the challenge of measuring them over the relevant time interval. The key anthropometric determinant of impending wasting, stunting, and mortality appears to be how far below the normal range the child’s weight is, rather than how they reached that position.

## Author contributions

The authors’ responsibilities were as follows – CMW, FP-R, FKH: designed the study and jointly wrote the first draft; FP-R, FKH: analyzed the data; RB, PA, SZ: provided the data, advised on interpreting the data and critically revised the manuscript; and all authors: read and approved the final version of the manuscript.

## Conflict of interest

The authors report no conflicts of interest.

## Funding

This analysis received no specific funding. The individual cohorts were funded from a wide range of sources: Lungwena Child Survival Study: Academy of Finland, Emil Aaltonen Foundation, Foundation for Pediatric Research, Medical Research Fund of Tampere University Hospital, the Research Foundations of Mannerheim League for Child Welfare, and the University of Tampere. Africa Center Vertical Transmission Study: Wellcome Trust UK 063009/Z/00/2. Lahore longitudinal study: Swedish Agency for Research Cooperation with Developing Countries (SAREC) and King Edward Medical College, Lahore, Pakistan.
