# Supplementary material for: Weight Velocity in Addition to Latest Weight Does Not Improve the Identification of Wasting or the Prediction of Stunting and Mortality: A Longitudinal Analysis Using Data from Malawi, South Africa, and Pakistan
Source: J Nutr. 2024 Jun 25;154(8):2583–9. doi: 10.1016/j.tjnut.2024.06.011 (PMC11375462; doi:10.1016/j.tjnut.2024.06.011)
Supplement: Multimedia component 1 [file mmc1.docx]

**Supplementary Materials**

**Weight velocity in addition to latest weight does not improve the identification of wasting, or the prediction of stunting and mortality: a longitudinal analysis using data from Malawi, South Africa, and Pakistan.**

Charlotte M Wright, et al.

Supplementary Table 1. Interaction of age group (≤ vs. > 6 months) and weight velocity with subsequent wasting

|  | Ratio of RR (95% CI) | p-value |
| --- | --- | --- |
| Model 1 |  |  |
| Wasting | 1.32 (1.10-1.59) | 0.003 |
| Model 2 |  |  |
| Wasting | 0.77 (0.58-1.03) | 0.081 |
| WAZ | 0.91 (0.84-0.98) | 0.01 |
| Model 3 |  |  |
| Wasting | 1.49 (1.23-1.80) | <0.001 |
| WVZ_2_ | 1.07 (1.02-1.13) | 0.007 |
| Model 4 |  |  |
| Wasting | 0.78 (0.58-1.04) | 0.091 |
| WAZ | 0.88 (0.81-0.95) | 0.001 |
| WVZ_2_ | 1.06 (1.01-1.12) | 0.02 |

The ratio of RR is the multiplicative interaction of age group and attained weight and weight velocity on wasting risk. A value above 1 indicates the HR is weaker (closer to 1) in the older age group. WAZ: weight-for-age z-score; WVZ: Weight velocity z-score.

Supplementary Table 2. Interaction of age group (≤ vs. > 6 months) and attained weight and weight velocity with subsequent stunting

|  | Ratio of RR (95% CI) | p-value |
| --- | --- | --- |
| Model 1 |  |  |
| Stunting | 0.92 (0.84; 1.01) | 0.077 |
| Model 2 |  |  |
| Stunting | 1.03 (0.91; 1.17) | 0.674 |
| WAZ | 1.05 (1.01; 1.10) | 0.016 |
| Model 3 |  |  |
| Stunting | 0.94 (0.86; 1.03) | 0.196 |
| WVZ_2_ | 1.06 (1.02; 1.09) | 0.001 |
| Model 4 |  |  |
| Stunting | 1.01 (0.89; 1.15) | 0.067 |
| WAZ | 1.04 (1.00; 1.09) | 0.857 |
| WVZ_2_ | 1.03 (0.99; 1.07) | 0.181 |

The ratio of RR is the multiplicative interaction of age group and attained weight and weight velocity on stunting risk. A value above 1 indicates the HR is weaker (closer to 1) in the older age group. WAZ: weight-for-age z-score; WVZ: Weight velocity z-score.

Supplementary Table 3. Interaction of age group (≤ vs. > 6 months) and attained weight and weight velocity with mortality

|  | Ratio of HR (95% CI) | p-value |
| --- | --- | --- |
| Not mutually adjusted |  |  |
| WAZ | 1.29 (1.003; 1.66) | 0.047 |
| WVZ_6_ | 1.27 (0.97; 1.66) | 0.084 |
| Mutually adjusted |  |  |
| WAZ | 1.16 (0.88; 1.51) | 0.290 |
| WVZ_6_ | 1.27 (0.95; 1.68) | 0.103 |

The ratio of HR is the multiplicative interaction of age group and attained weight and weight velocity on mortality risk. A value above 1 indicates the HR is weaker (closer to 1) in the older age group. WAZ: weight-for-age z-score; WVZ: Weight velocity z-score.
